# Real-time 3D Photoacoustic Visualization System with a Wide Field of View for Imaging Human Limbs

**DOI:** 10.12688/f1000research.16743.2

**Published:** 2019-02-07

**Authors:** Kenichi Nagae, Yasufumi Asao, Yoshiaki Sudo, Naoyuki Murayama, Yuusuke Tanaka, Katsumi Ohira, Yoshihiro Ishida, Atsushi Otsuka, Yoshiaki Matsumoto, Susumu Saito, Moritoshi Furu, Koichi Murata, Hiroyuki Sekiguchi, Masako Kataoka, Aya Yoshikawa, Tomoko Ishii, Kaori Togashi, Tsuyoshi Shiina, Kenji Kabashima, Masakazu Toi, Takayuki Yagi

**Affiliations:** 1 Medical Imaging System Development Center, Canon Inc., 3-30-2 Shimomaruko, Ohta-ku, Tokyo, 1468501, Japan; 2 ImPACT Program, Japan Science and Technology Agency, K’s Gobancho, 7, Gobancho, Chiyoda-ku, Tokyo, 1020076, Japan; 3 Department of Breast Surgery, Graduate School of Medicine, Kyoto University, 54 Shogoin-Kawaharacho Sakyo-ku, Kyoto, 6068507, Japan; 4 Healthcare Ultrasound R&D Center, Hitachi, Ltd., 3-1-1, Higashikoigakubo, Kokubunji-shi, Tokyo, 1850014, Japan; 5 Research & Development Center, Japan Probe Co., Ltd., 1-1-14, Nakamura-cho, Minami-ku, Yokohama, Kanagawa, 2320033, Japan; 6 Department of Dermatology, Graduate School of Medicine, Kyoto University, 54 Shogoin-Kawaharacho Sakyo-ku, Kyoto, 6068507, Japan; 7 Department of Plastic and Reconstructive Surgery, Graduate School of Medicine, Kyoto University, 54 Shogoin-Kawaharacho Sakyo-ku, Kyoto, 6068507, Japan; 8 Department of Orthopaedic Surgery, Graduate School of Medicine, Kyoto University, 54 Shogoin-Kawaharacho Sakyo-ku, Kyoto, 6068507, Japan; 9 Department of Diagnostic Imaging and Nuclear Medicine, Graduate School of Medicine, Kyoto University, 54 Shogoin-Kawaharacho Sakyo-ku, Kyoto, 6068507, Japan; 10 Department of Human Health Science, Graduate School of Medicine, Kyoto University, 53 Shogoin-Kawaharacho Sakyo-ku, Kyoto, 6068507, Japan

**Keywords:** photoacoustic imaging, optoacoustic, hemispherical detector array, laser, limb, haemoglobin oxygen saturation, motion pictures, blood vessel

## Abstract

**Background** *: *A breast-specific photoacoustic imaging (PAI) system prototype equipped with a hemispherical detector array (HDA) has been reported as a promising system configuration for providing high morphological reproducibility for vascular structures in living bodies.

**Methods** *: *To image the vasculature of human limbs, a newly designed PAI system prototype (PAI-05) with an HDA with a higher density sensor arrangement was developed. The basic device configuration mimicked that of a previously reported breast-specific PAI system. A new imaging table and a holding tray for imaging a subject's limb were adopted.

**Results** *: *The device’s performance was verified using a phantom. Contrast of 8.5 was obtained at a depth of 2 cm, and the viewing angle reached up to 70 degrees, showing sufficient performance for limb imaging. An arbitrary wavelength was set, and a reasonable PA signal intensity dependent on the wavelength was obtained. To prove the concept of imaging human limbs, various parts of the subject were scanned. High-quality still images of a living human with a wider size than that previously reported were obtained by scanning within the horizontal plane and averaging the images. The maximum field of view (FOV) was 270 mm × 180 mm. Even in movie mode, one-shot 3D volumetric data were obtained in an FOV range of 20 mm in diameter, which is larger than values in previous reports. By continuously acquiring these images, we were able to produce motion pictures.

**Conclusion** *: *We developed a PAI prototype system equipped with an HDA suitable for imaging limbs. As a result, the subject could be scanned over a wide range while in a more comfortable position, and high-quality still images and motion pictures could be obtained.

## Introduction

Blood vessels are important for the delivery of oxygen and nutrients to the entire body. Vascular imaging plays an important role in the diagnosis of congenital vascular abnormalities, diseases of the blood vessels themselves, and angiogenesis associated with diseases such as cancer ^1^. Various modalities are used in clinical practice to diagnose diseases by imaging blood vessels. These modalities have some disadvantages, such as the need for contrast medium, exposure to X-ray radiation, or expensive equipment, such as magnetic resonance imaging (MRI) systems. On the other hand, blood flow imaging is also possible with Doppler ultrasound (US), which does not require contrast medium and is not invasive; however, its spatial resolution is limited.

Photoacoustic tomography (PAT) can be used to visualize blood vessels with high resolution noninvasively ^2^. In particular, systems ^3– 10^ using a hemispherical detector array (HDA) as a sensor can reconstruct blood vessel images with good 3D reproducibility. We produced a prototype breast-specific PAT system (PAI-03, 04) ^3, 5– 10^ and used it to conduct clinical research by scanning breast cancer patients in the prone position and visualize tumor-related blood vessels caused by breast cancer. The probe arrangement of the HDA was designed to solve the "limited view problem" ^11^ that causes PAT image degradation. This probe arrangement surrounds the measurement target with a much wider solid angle than that of a linear probe with the PAT apparatus ^12– 17^.

The above studies demonstrated the acquisition of high-resolution 3D still images with a hemispherical probe over a large area by wide-range scanning of the measurement area.

We also scanned the healthy blood vessels in various body parts other than the breasts with these devices and reported the potential clinical usefulness of this approach ^6– 10^. By analyzing the blood vessels in the palms of healthy volunteers, we showed that the tortuosity of blood vessels increases with age, suggesting the possibility of assessing the risk of diseases such as arteriosclerosis and other lifestyle diseases. In addition, by imaging perforators in the anterolateral thigh (ALT), this method was demonstrated to be effective for the preoperative planning of ALT free flaps. Although the possibility of this system being useful for imaging blood vessels in contexts other than breast cancer, especially in limbs, has been suggested, there were some related issues, such as the subject having to assume an awkward position and the measurement range being limited due to the shape of the imaging table, which was originally designed for studying breast cancer.

With the aim of imaging blood flow in limbs, we have developed a new PA imaging (PAI) system prototype (PAI-05) equipped with an HDA that generally follows a conventional design. Furthermore, the system is capable of real-time 3D imaging. This paper outlines the device configuration and introduces examples of biological images obtained using this device.

## Methods

### Device configuration

**Figure 1.**
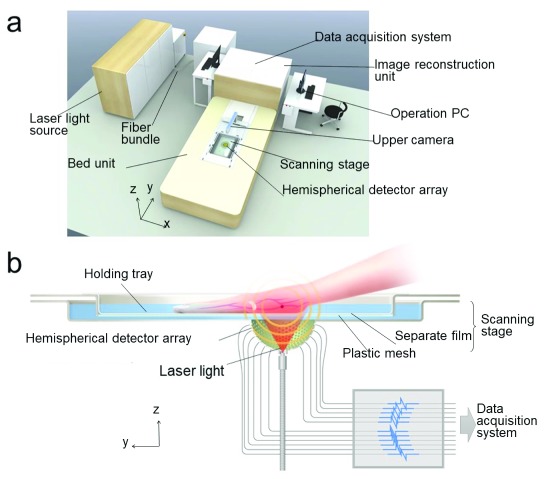
Schematic illustration showing the PAI-05 system configuration and the principle of data acquisition. ( **a**) Top view of the PAI-05 system, which consists of a laser light source, a data acquisition unit, an image reconstruction unit, a bed unit, and an operation PC. A scanning stage with an hemispherical detector array (had) set in the bed unit. Oscillating laser light is delivered to the bottom of the HDA through a fiber bundle. A subject can be observed by an upper camera. ( **b**) Cross-sectional view of the HDA and holding tray cut at the center during the photoacoustic (PA) measurement. The generated PA wave is received by the HDA through the holding tray, which consists of plastic mesh and a separate film., The PA signal is then sent to the data acquisition system (DAS).

The basic design of PAI-05, which was developed in cooperation with Canon, Inc., Hitachi, Ltd., and Japan Probe Co., Ltd., mimics that of the conventional prototype ^3, 5– 10^ equipped with an HDA that receives the PA signal. An overall image of PAI-05 is shown in Figure 1a. The PAI-05 system consists of a bed unit, including an HDA and a scanning stage, a light source, a fiber bundle, a data acquisition system (DAS), a real-time image reconstruction unit, and an operation personal computer (PC). In this paper, the horizontal plane is the *x*- *y* plane, and the x and y axes are the short and long axes of the bed unit, respectively. The *z* axis is the vertical axis.

When scanning, the body part of the subject is inserted into the holding tray on the bed unit ( Figure 1b). The previous prototypes adopted hemispherical holding cup shapes because they were designed for imaging breasts, but in the PAI-05, the holding tray has a flat bottom to facilitate imaging of the hands and feet. In addition, the bed size was enlarged so that a subject could be measured in various postures. To maintain the flat shape of the bottom surface, it was supported by plastic mesh, and a sheet made of polyethylene terephthalate (PET) film was used on the mesh to separate the acoustic matching water on the HDA from the tray water, which allowed the subject to be immersed ( Supplementary Video 1).

**Figure 2.**
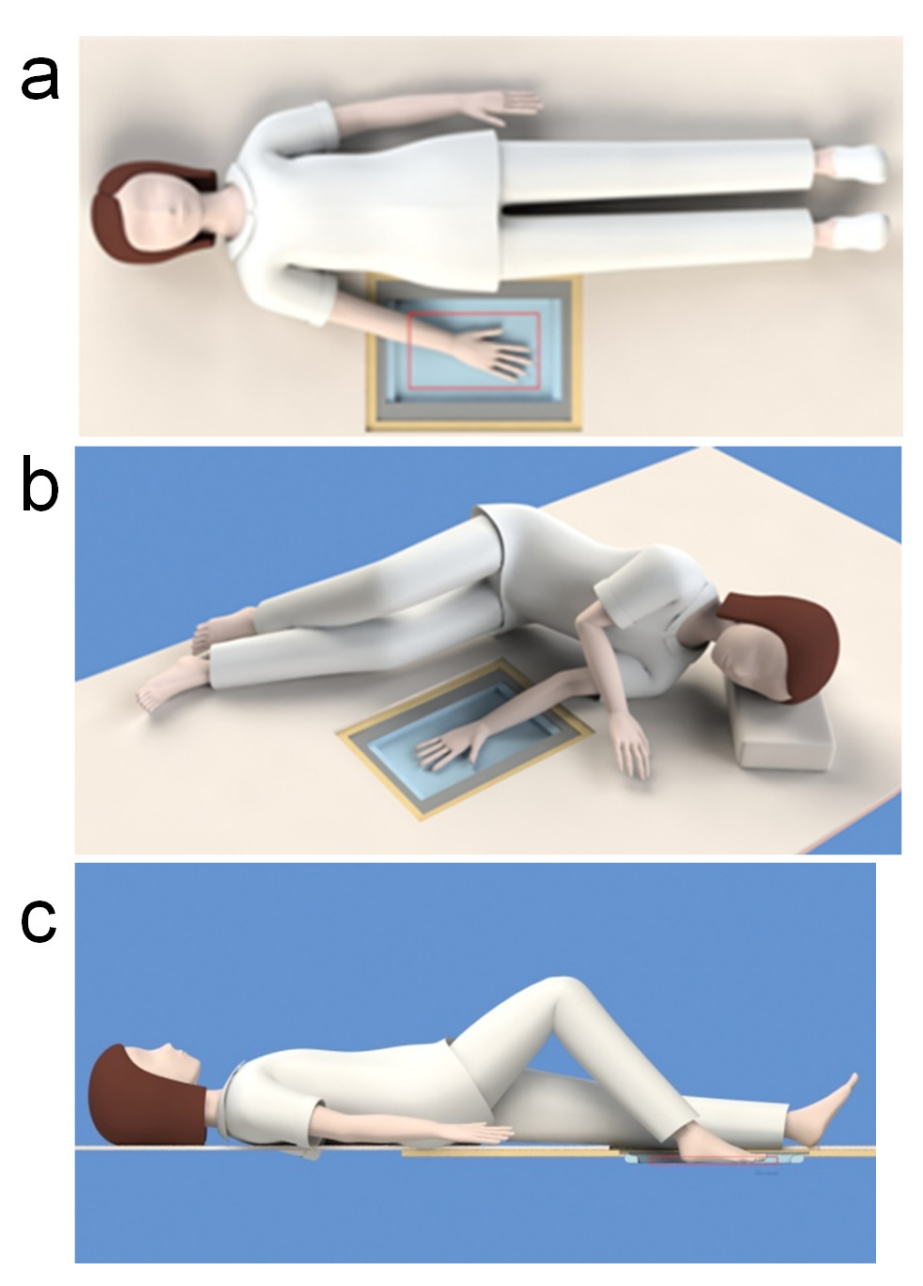
Schematic diagram illustrating the postures of a subject during photoacoustic imaging (PAI). ( **a**) Example posture assumed during the capture of a PA image of a palm. The subject lies down on the side of the holding tray and inserts the hand into the tray naturally. ( **b**) Example posture used for imaging the back of the hand. ( **c**) Example posture used for imaging the sole of the foot.

Conventionally, the subject's breast was inserted in the hemispherical holding cup while the subject was in the prone position. On the other hand, in the PAI-05 system, to make it easier to scan limbs, the subject lies next to the holding tray filled with water for acoustic matching. This configuration allows the subject to be scanned in a more comfortable posture than that required for imaging the palm with the previous prototype ^6^. Figures 2a–c show the posture assumed when scanning the palm, back of the hand, and sole of the foot, respectively. The size of the holding tray was 529 × 259 mm, and the depth was 29.4 mm. The bed size was 1.4 m along the *x* axis, 2.7 m along the *y* axis and 0.48 m along the *z* axis.

In the conventional breast-specific PAI system, the object to be imaged was nearly round, so it was spirally scanned to form a PA image of circular scanning range ^3, 5– 10^. On the other hand, in the case of limbs, since the measurement target is often noncircular with the direction of the bone as the major axis, the scanning range is rectangular. To avoid a decrease in speed when changing the scanning direction at the vertex, the corners were scanned in a manner producing a smooth locus ( Supplementary Video 2).

The measurement target was immersed in a holding tray, and the scanning unit containing the HDA was placed under the bed. The maximum still image measurement area was 270 mm × 180 mm, and the measurement time was proportional to the imaging area. The measurement area size and measurement time are shown in Table 1.

**Table 1.** The relationship between scanning area and scanning time.

Mode	Scan size (x × y)	Scan time
Still mode	180 mm × 270 mm	573 s
	180 mm × 135 mm	315 s
	100 mm × 50 mm	106 s
	50 mm × 100 mm	106 s
	40 mm × 40 mm	58 s
Movie mode	20 mmΦ	-

**Figure 3.**
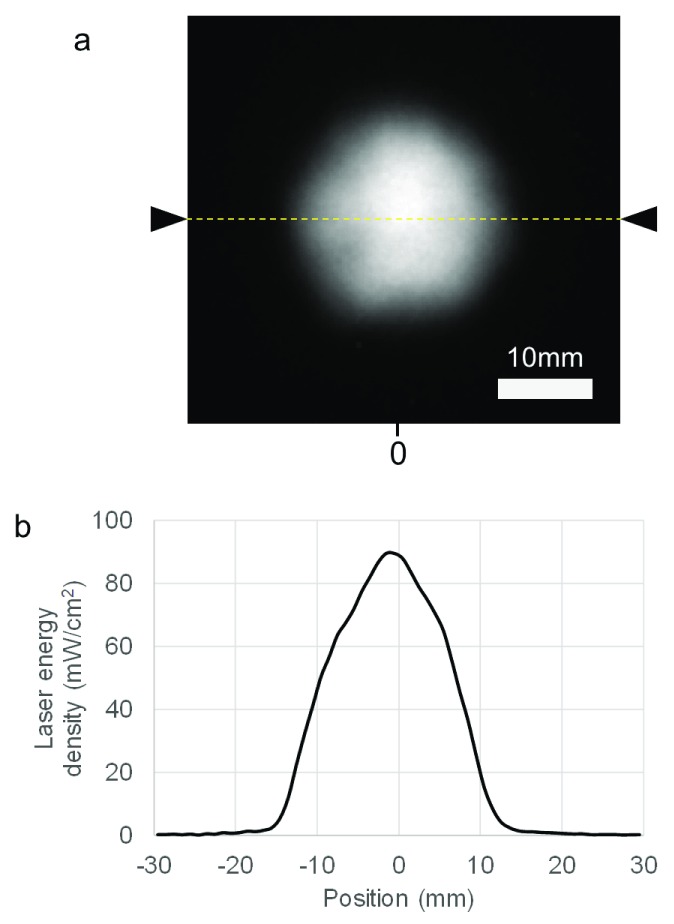
Example of actual results of the laser irradiation profile. ( **a**) Photograph of the laser irradiation profile. This picture was taken by a digital camera while a diffusion film was on the bottom of the tray. ( **b**) Energy density distribution of the laser light along the dotted line in Figure 3a.

A giant-pulse laser beam with a pulse width of less than 20 ns was directed upward along the z axis from the emission end of the fiber bundle on the bottom surface of the HDA. One laser system per wavelength (Lotis TII, Belarus) was used. Using two sets of lasers, fiber bundles were used to guide laser light to the HDA, and alternate irradiation at the two wavelengths was performed. Part of the fiber bundles was pulled out to detect and synchronize the signal reception timing at the DAS unit. The diverging lens located at the base of the HDA conically spread the light to a diameter of 24.8 mm at the surface of the holding tray. Figures 3a–b show the laser profiles.

One laser irradiated light at a repetition frequency of 10 Hz. Each wavelength could be selected from the wavelength range of 750 to 850 nm using an optically pumped Ti:Sa laser and a Q-switched Nd:YAG laser. The wavelength could be set at steps of 1 nm.

To form a hemoglobin oxygen saturation distribution image, wavelengths of 756 and 797 nm were selected, as in the conventional case; 756 nm is the maximum point of the absorption coefficient of deoxygenated hemoglobin, and 797 nm is the isosbestic point of oxyhemoglobin and deoxygenated hemoglobin.

In PAI-05, alternate irradiation ^10, 18^ was performed using two different laser systems every 50 ms. The exposure was set to be smaller than the maximum permissible exposure recommended by ANSI at any wavelength. When two lasers were set to the same wavelength, they could irradiate at 20 Hz.

**Figure 4.**
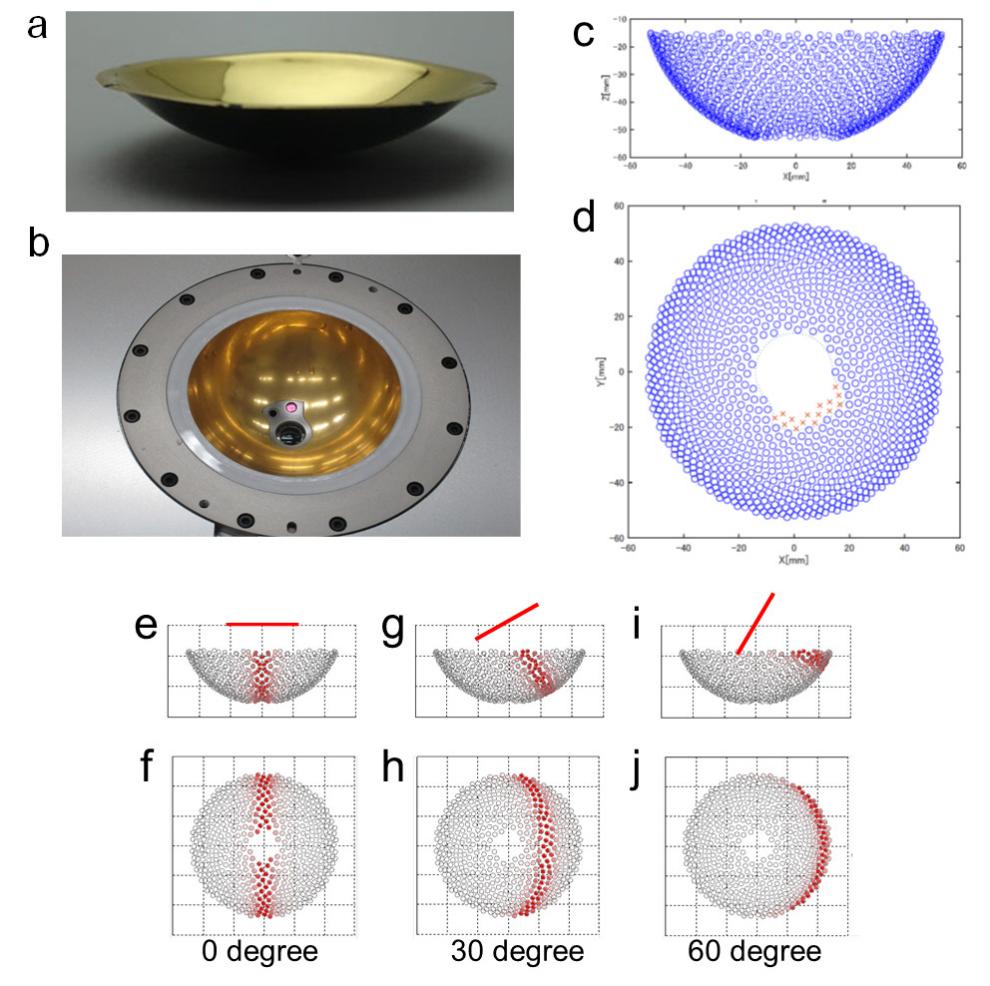
Pictures and schematic illustration of the design and properties of the hemispherical detector array (HDA). ( **a**) Photograph of a film-shaped ultrasound sensor used in the HDA. Thereafter, a lead wire was connected to a predetermined position to form a sensor array of 1024 channels. ( **b**) Photograph of the completed HDA module. The large hole in the center is a laser emission hole. The drainage outlet and lower camera are installed next to the laser emission hole at the bottom of the HDA. ( **c**) Side view of the probe array. Sensor positions are indicated as blue circles. ( **d**) Top view of the probe array. Sensors are placed according to the 3D Fibonacci grid, excluding areas indicated by blank or orange X marks, where the laser emission hole, drainage outlet and lower camera are located. ( **e**)–( **j**) Diagrams schematically showing the photoacoustic (PA) signal intensities reaching each sensor element when a rod-shaped phantom is installed directly above the HDA and a PA signal is generated. Pure red color indicates a strong PA signal arriving at a sensor, and white color indicates no PA signal. ( **e**) Side view of the PA signal intensity distribution on the HDA when a rod-shaped phantom is horizontally installed and a signal reaches the sensor. ( **f**) Top view of the PA signal intensity distribution under the same conditions as in Figure 4e. ( **g**) Side view of the PA signal intensity distribution on the HDA when a rod-shaped phantom is installed inclined at an angle of 30 degrees from the horizontal and a signal reaches the sensor. ( **h**) Top view of the PA signal intensity distribution under the same conditions as in Figure 4g. ( **i**) Side view of the PA signal intensity distribution on the HDA when a rod-shaped phantom is installed inclined at an angle of 30 degrees from the horizontal and a signal reaches the sensor. ( **j**) Top view of the PA signal intensity distribution under the same conditions as in Figure 4g.

We used an HDA manufactured by Japan Probe Co., Ltd., for the PA signal detector. An ultrasonic flexible array probe ^19^ using a 1–3 composite piezoelectric transducer made of lead zirconate titanate (PZT) was adopted to arrange 1024 channel sensors on a 55-mm-radius hemisphere of epoxy resin. By applying this device to the hemispherical film, a piezoelectric vibrator with a three-layer structure consisting of a protective layer, a composite resonator, and a damper material was formed ( Figure 4a). Next, the lead wire was mounted on the hemispherical film-like piezoelectric vibrator using a fine soldering technique, thereby forming a 1024-channel element in a spherical shape, thus completing the HDA ( Figure 4b). The diameter of the single circular element was 2 mm. On the hemispherical sensor, the sensor element was arranged according to a 3D Fibonacci grid ^20^, as shown in Figures 4c–d. With this arrangement, the receiving element density per solid angle became nearly uniform, and artifacts generated by image reconstruction could be suppressed. The center frequency and the fractional bandwidth of the device were 3.34 MHz and over 85%, respectively. We set the amplification rate to 48 dB in this study. The average conversion efficiency was 1.9 mV/kPa at 2.5 MHz. The noise equivalent pressure (NEP) measurement was examined using a low-pass filter with a cut-off frequency of 5 MHz, and the NEP, the effective value without signal averaging, of this system was 3.42 Pa.

### Data acquisition

The two video images, one from the lower camera (not shown) installed on the bottom of the HDA, and one from the upper camera, as shown in Figure 1a, at the top were displayed on the screen during scanning.

The PA signal was received by the HDA, and the signal data were transferred to the DAS, which was manufactured by Hitachi, Ltd. The DAS amplified the PA signal of 1024 channels at the time of light irradiation from the laser unit, simultaneously sampled 1024 channels at 60 MHz and 12 bits, and converted the signals into digital data. The digital data were sequentially transferred to the image reconstruction PC.

Real-time image reconstruction could be performed during scanning, and the reconstructed PA image was displayed on the operation PC.

The Digital Imaging and Communications in Medicine (DICOM) image data reconstructed inside the PAI-05 system based on the DICOM standard (version 3.0) could be automatically transferred to the DICOM image server of Kyoto University Hospital.

The PAI-05 system could perform two types of imaging: one in still mode, in which a large area could be imaged by scanning with the HDA; and another in movie mode, in which a specific place was irradiated with laser light to repeatedly obtain updated PA images of that area.

In still mode, images could be acquired with the scanning sizes shown in Table 1, and the PA images were generated by scanning with the HDA and taking the average of more than 24 scans within the imaging range. For PAI-05, imaging ranges of 40 mm × 40 mm, 50 mm × 100 mm, 100 mm × 50 mm, 135 mm × 180 mm, and 270 × 180 mm could be selected. The scanning stage was controlled to move in a rectangular spiral shape.

Two types of HDA scanning operations were available in movie mode. One type was denoted as simple movie (SM) mode, in which the PA image is updated by repeatedly irradiating one spot with laser light while the HDA remained stationary on the spot. The other type was denoted as fluctuation movie (FM) mode, in which imaging was performed during continuous minute rotational motion. In any movie mode, it was possible to acquire an accumulation of images over time for a reconstructed image according to the number of times of laser light was irradiated. In FM mode, the scanning operation occurred at 3 rotation/sec such that the center of the HDA would follow the trajectory of a circle with a diameter of 3 mm ( Supplementary Video 3).

### Imaging method

We used universal back-projection (UBP) ^21^ for PA image reconstruction. When calculating the absorption coefficient from the initial photoacoustic pressure, it is necessary to perform the light intensity correction. In this paper, however, we did not carry out it and limited the discussion within the qualitative consideration. An image was created for each shot of the laser and recorded in movie or still mode. The former method yielded real-time motion pictures, and the latter method yielded a high-quality still image by accumulating scanned images to construct an image with a wide field of view (FOV). The volume shape of the reconstructed image per irradiation, which was occurred every 50 ms, was a circular cylinder. The base was a circle with a diameter of 20 mm in the *x*- *y* plane, and the height was 30 mm in the *z* direction. These image reconstructions were realized in real time by pipeline-processing data transfer and reconstruction operations using five graphics processing units (GPUs, FirePro S9150, Advanced Micro Devices, Inc., USA). The state of reconstruction during scanning was displayed on the operation PC. The voxel size when displaying the reconstruction on the display in real time was 0.1 mm in the *x*, *y,* and *z* directions.

The limitation of this system is the “limited view problem” ^11^, which is commonly found in other PA systems. Adopting the HDA in the PAI-05 system remarkably alleviates the problem compared to the use of handheld (HH) devices. Nonetheless, the “limited view problem” persists in the PAI-05 system. Figures 4e–j show the result of simulating PA waves generated from rod-like subjects placed at the center of the sensor. The angle of each rod-like subject was set to 0, 30, and 60 degrees, as illustrated in Figures 4e–f, 4g–h, and 4i–j, respectively. When the rod-like subject was set to the horizontal plane or when the inclination was approximately 30 degrees, the signal could be fully received by the HDA. If the subject was set to an inclination of 60 degrees, the PA signal was received at almost the top of the HDA. Although our simulation showed that signals could be received from subjects up to a tilt to 70 degrees, image reconstruction cannot be achieved with a rod-like absorber that is more inclined, i.e., close to vertical. It is considered that many vessels underlying the limbs targeted by the PAI-05 system are largely parallel to the surface of the skin in general, but careful attention is required for analysis.

Ideally, oxygen saturation can be calculated using equation (1) if the absorption coefficients of two wavelengths can be correctly obtained.

${\mathrm{SO}}_{2}\;=\;\frac{\left[{\mathrm{HbO}}_{2}\right]}{\left[{\mathrm{HbO}}_{2}]+[\mathrm{Hb}\right]}\;=\;\;\frac{\frac{\mu_{a}^{\lambda_{2}}(r)}{\mu_{a}^{\lambda_{2}}(r)}.\;\varepsilon_{\mathrm{Hb}}^{\lambda_{1}}–\varepsilon_{\mathrm{Hb}}^{\lambda_{2}}}{\varepsilon_{\Delta \mathrm{Hb}}^{\lambda_{2}}–\frac{\mu_{a}^{\lambda_{2}}(r)}{\mu_{a}^{\lambda_{1}}(r)}.\varepsilon_{\Delta \mathrm{Hb}}^{\lambda_{1}}}\;(1)$

where *λ*_1_ and *λ*_2_ represent wavelengths of 756 and 797 nm, respectively; *r* is the position to be calculated; *ε*_*Hb*_ is the molar extinction coefficient of deoxyhemoglobin; and *ε*_Δ *Hb*_ is the difference in the molar extinction coefficients between deoxyhemoglobin (Hb) and oxyhemoglobin (HbO _2_).

In real situations, since ideal hemoglobin oxygen saturation (SO _2_) cannot be obtained due to various error factors, we refer to the parameter obtained by the two wavelengths as the S-factor ^15^. As described previously ^10^, the relation of the magnitude of SO _2_ and the S-factor is maintained within the range where the amount of irradiated light can be regarded as the same as that in a neighboring region.

Calculation of the S-factor was not performed by the PAI-05 system; instead, after data acquisition, PA signal data were copied to another PC, and the calculation were performed off-line. A weighted S-factor ^15^ was used for image display by producing a weighted image with a signal intensity of 797 nm. To calculate these S-factors and display PA images, we used a PAT-dedicated viewer named Kurumi [version 3.91 ^22^].

Kurumi is equipped with a body surface detection function that uses cloth simulation ^23^, which makes it easier to analyze deeper areas by deleting unnecessary image information, such as that related to subcutaneous vein networks, as necessary. To detect the position of the body surface, an image obtained at 797 nm was mainly used.

### Imaging subjects

**Figure 5.**
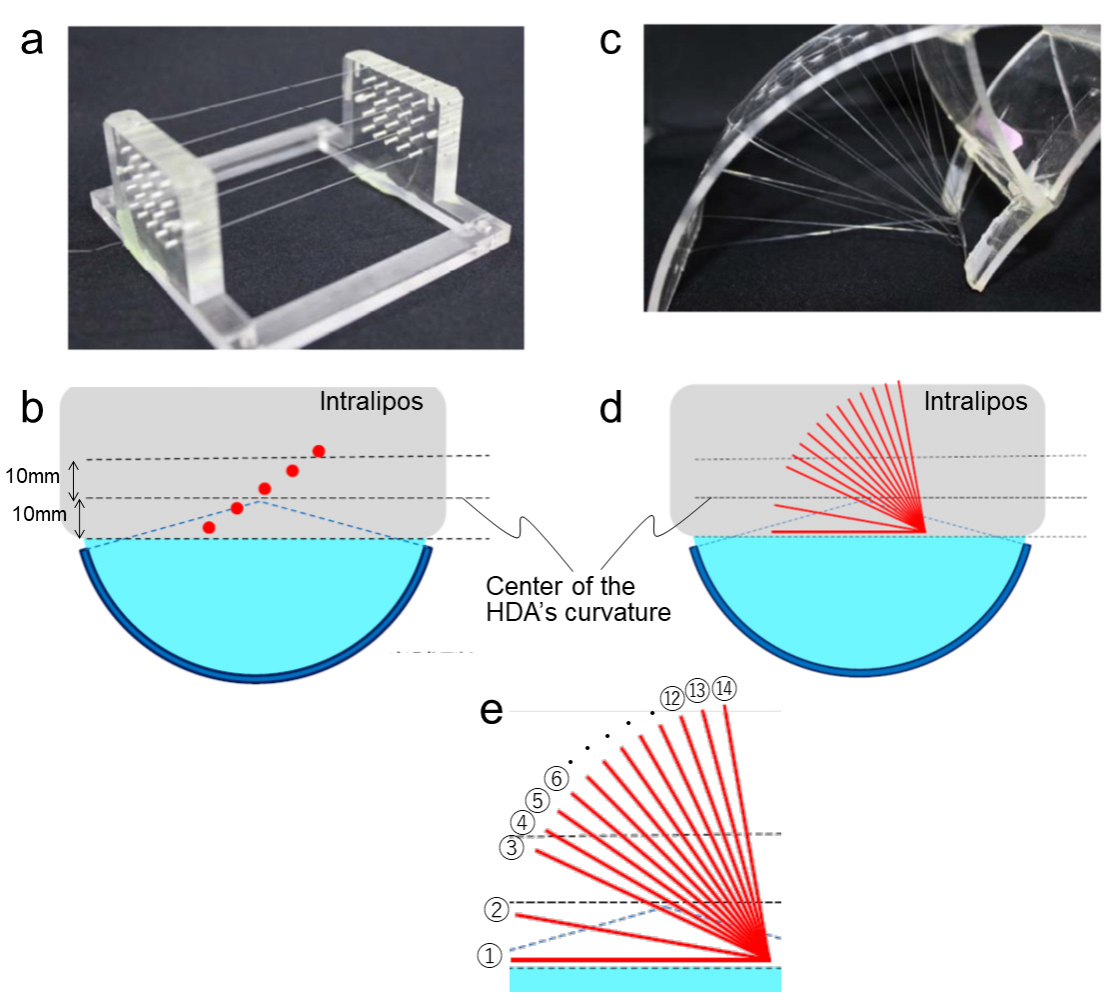
Pictures and schematic illustration of phantoms. ( **a**) Photograph of a phantom used for evaluating the penetration depth of the PAI-05 system. ( **b**) Schematic illustration of the phantom shown in Figure 5a. ( **c**) Photograph of a phantom used for evaluating the viewing angle of the PAI-05 system. ( **d**) Schematic illustration of the phantom shown in Figure 5c. ( **e**) Enlarged image of schematic in Figure 5d.

The phantom used in this study was as follows. Surgical thread (11-0, thickness, 10–19 μm, Monosof, Medtronic plc, Ireland) was used for a line spread function (LSF) evaluation (not shown). The thread was placed parallel to the horizontal plane at a depth of a few mm closer to the tray bottom from the center of curvature of the HDA. Figures 5a–b show the phantom structure that was used to evaluate the penetration depth. A wire phantom (diameter, 0.3 mm, *μ _a_*: 0.22 mm ^-1^) mimicking the light absorption coefficient of blood at 797 nm was placed in Intralipos Injection 20％ (Otsuka Pharmaceutical Co., Ltd., Japan) diluted to 1% concentration ( *μ _a_*: 0.0022 mm ^-1^, $\mu_{s}^{'}$: 0.921 mm ^-1^); *μ _a_* is the absorption coefficient, and $\mu_{s}^{'}$ is the equivalent scattering coefficient. Five wire phantoms were installed at positions of 0, 5, 10, 15 and 20 mm along the *z* axis from the bottom of the tray.

To evaluate the allowance of the visualization range of the “limited view problem,” we used wire phantoms ( Figures 5c–d) installed in Intralipos at an angle to the sensor scanning plane or the *x*- *y* plane.

To simply evaluate the wavelength dependence of the PA signal intensity, a line written on white office paper with four colors of oil-based ink pen (black, red, green and blue) was used. A V-680 spectrophotometer (JASCO Co., Japan) was used to evaluate the reflectance spectral characteristics of the office paper and oil-based ink written on the paper.

For living body measurements, one healthy male subject was recruited. He was 173 cm tall, his foot was 25.5 cm long, and he was in his fifties. The hair of the imaged limb was cut beforehand with hair clippers, but when the remaining hair roots interfered with the analysis, the hair root data was excluded using the body surface detection method described above. This subject was registered as a healthy volunteer in a control group of an exploratory clinical study for examining the vascular condition of patients with a skin disease using the same PAI-05 system. In the current study, biological data from the clinical study were only utilized for the presentation of imaging examples. The results of the skin disease analyses will be reported in the near future.

### Ethics

The present study was approved by the Ethics Committee of the Kyoto University Hospital (UMIN 000022767), and written informed consent was obtained from the subject. This study was conducted in accordance with the Declaration of Helsinki.

## Results

### Phantom experiments

**Figure 6.**
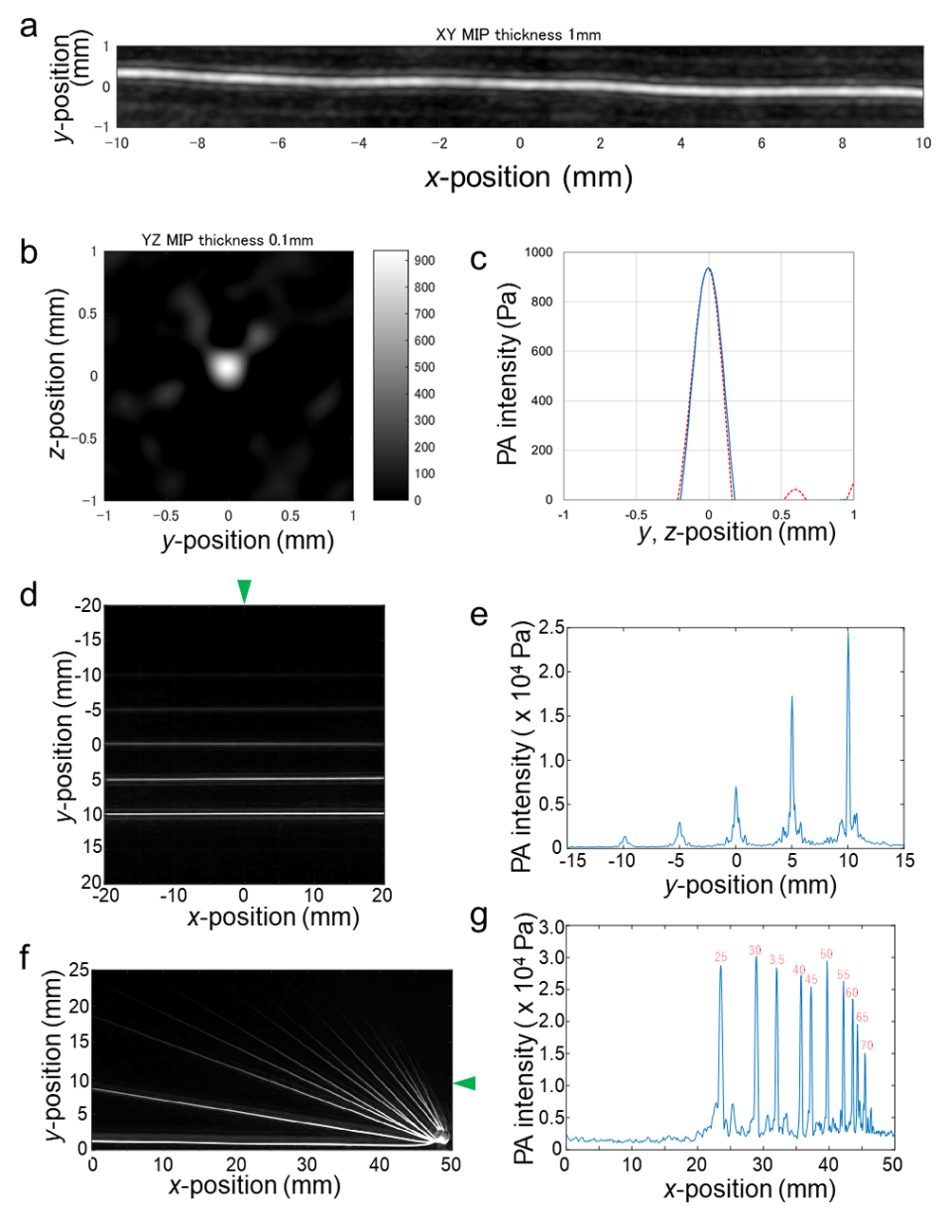
Experimental results of phantoms for the line spread function (LSF) ( **a**– **c**), penetration depth ( **d**– **e**) and viewing angle ( **f**– **g**). ( **a**) Maximum intensity projection (MIP) image on the x-y plane obtained by scanning the phantom made of a thin thread. ( **b**) Cross-sectional view of Figure 6a as viewed from the y-z plane. ( **c**) Line profile of photoacoustic (PA) intensity at the centerline of Figure 6b. ( **d**) MIP image of a phantom for penetration depth evaluation. ( **e**) Profile of PA intensity at x=0 in Figure 6d. ( **f**) MIP image of a phantom for viewing angle evaluation. ( **g**) PA intensity diagram for each phantom with different angles.

The performance of the system was evaluated using phantoms. The results are shown in Figure 6. The results obtained with the surgical thread phantom are shown in Figures 6a–c. As a result of evaluating the LSF, the full width at half maximum (FWHM) in the direction parallel to the HDA scanning plane was 0.21 mm, and the FWHM for the *z* axis was also 0.21 mm.

Figures 6d–e show the results of the depth performance evaluation. A wire phantom at a depth of 20 mm in the Intralipos could be visualized with a signal-to-noise ratio (SNR) of the maximum intensity projection (MIP) image of approximately 8.5, where the noise value was the average of the signal background level. The results of evaluating the “limited view” are shown in Figures 6f–g. It was experimentally shown that a 70-degree wire phantom could be visualized. A 75-degree wire was difficult to observe.

The image quality of the movie mode was evaluated using a wire phantom while changing the number of PA images averaged in both SM and FM modes.

**Figure 7.**
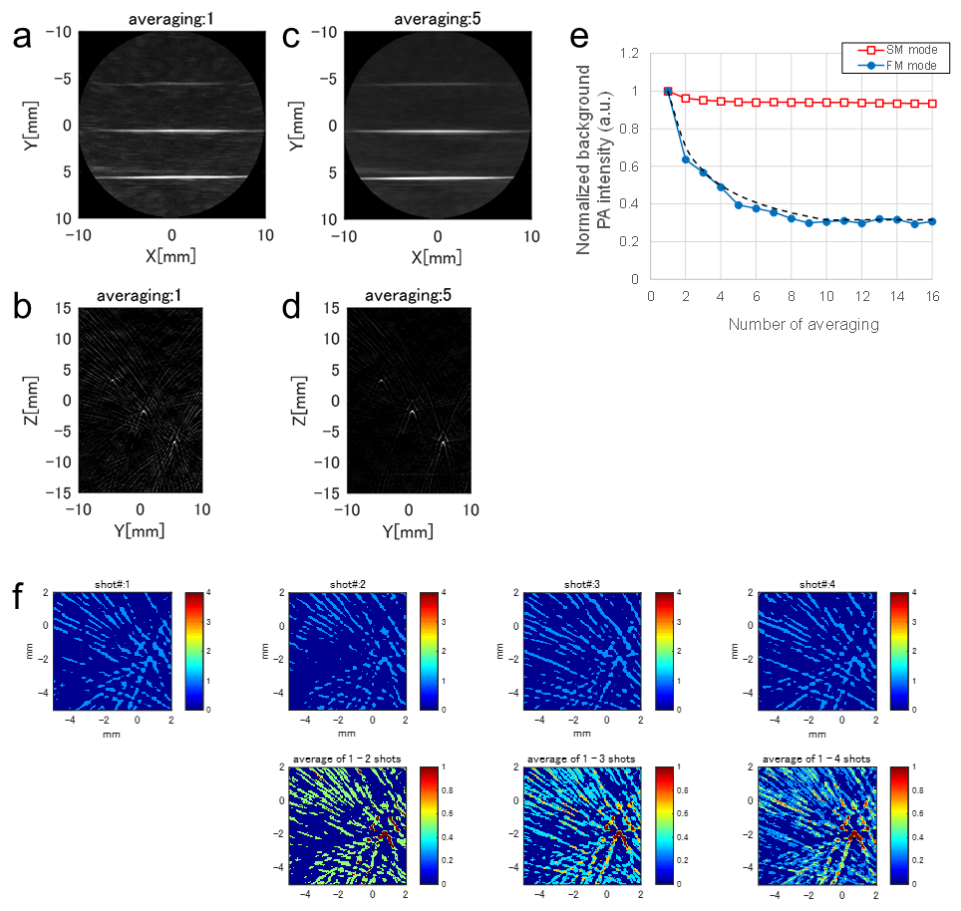
Phantom evaluation results obtained by changing the scan modes and N in movie mode. ( **a**) Maximum intensity projection (MIP) image of the x-y plane of only one shot (i.e., without averaging). ( **b**) MIP image of the y-z plane in Figure 7a. ( **c**) MIP image of the x-y plane with N=5 in fluctuation movie (FM) mode. ( **d**) MIP image of the y-z plane in Figure 7c. ( **e**) Graph showing the normalized background noise as a function of N. In SM mode, the noise level does not decrease even if N is increased, but the noise level decreases with 1/Route N in FM mode. However, the dependence on the number of averaged images disappears after N=10, and the noise remained nearly constant in FM mode. The dashed line indicates an approximate curve of N in FM mode, showing 1/Route N before the N=10 and a constant line after N=10. ( **f**) The upper row shows tomographic images of the y-z plane with only one shot for the first to the fourth images in FM mode. To facilitate the artifact analysis, binarization was performed; values 1/20 or more of the peak intensity was considered noise, and the intensity value of that position was set to 1; the intensity value of the remaining positions, where the noise was less than 1/20 of the peak intensity, was set to zero. From left to right, the lower row shows the images obtained in FM mode with N=2, N=3, and N=4. The intensity of the true signals of the phantom is almost 1, and the intensity of the artifact decreases as N increases.

Figures 7a–b show examples of images without averaging, and Figures 7c–d show examples of five images averaged in FM mode. There was an obvious difference in the background noise of the cross-sectional view of the phantom without averaging (i.e., an average of 1) and with averaging of 5 images, as shown in Figures 7b and 7d. To quantify the amount of noise, the background noise of data obtained in FM and SM mode was evaluated using the root mean square (RMS) value. The background was defined as inside the area ( *x*: 6 mm, *y*: 2.5 mm, *z*: 2.5 mm) where the wire phantom did not exist.

For the noise analysis, a plot of the background noise after normalization by the background noise without averaging was made as a function of the number of images averaged (N), as shown in Figure 7e. The background noise in FM mode decreased in proportion to $1/\sqrt{N}$ until N reached ten; furthermore, when N was 11 or more, the background noise did not decrease but remained nearly constant. Because the rotational scanning of the sensor in FM mode was 3 rotations/sec, the first data acquisition position coincided with the eleventh position.

In SM mode, reducing the background noise only slightly reduced the system noise with respect to N. The FM mode showed a greater noise reduction effect than the SM mode. To analyze the noise reduction effect in FM mode, Figure 7f was created. Each image in the upper row represents 1 shot at a different position in FM mode; binarization processing was performed with 1 as the part exceeding the 1/20 luminance value of the wire phantom contained in the screen and displayed. Among these areas, the noise area is a portion that expresses 1 even though no wire phantom subject exists in that area. The noise extends in a streak shape from the wire portion and is considered an image artifact. The lower row shows FM mode images obtained by averaging 2 shots, 3 shots, and 4 shots, respectively, from the diagram on the left. As the images overlapped after shifting the position of the streak artifact, the intensity of the noise could be reduced by averaging the whole images. Needless to say, the image averaging caused degradation of the temporal resolution. There was an obvious trade-off between temporal resolution and image quality in FM mode, and degradation of the temporal resolution occurred almost without improving image quality in SM mode.

**Figure 8.**
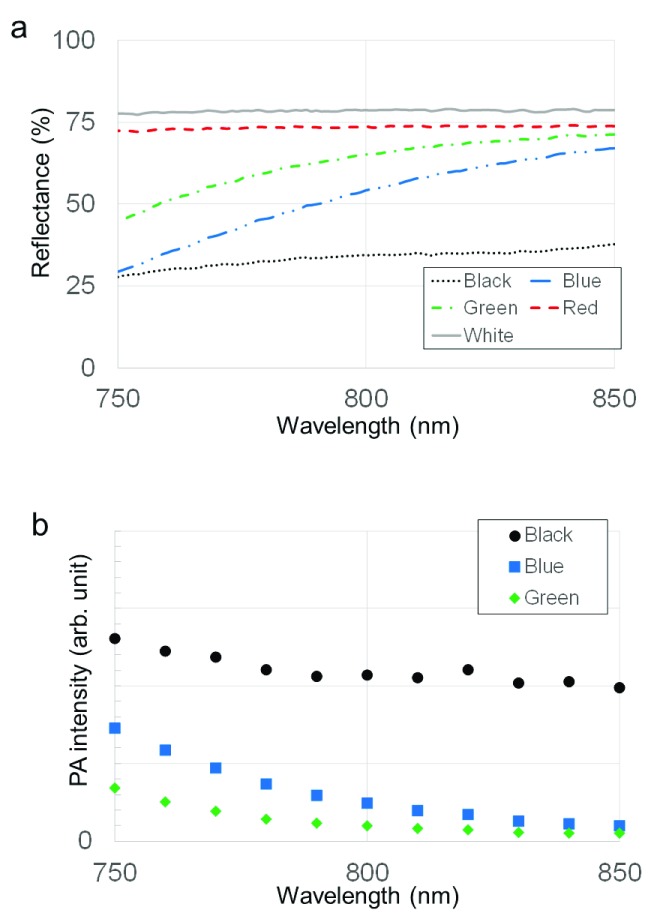
Results of simplified PA spectrum evaluation using office paper and colored oil-based ink. ( **a**) Diagram showing the reflectance spectra for oil ink on white office paper. In the legend, white indicates the reflectance of the office paper, and black, red, green, and blue indicate the reflectance of the ink on the office paper. ( **b**) Diagram showing the photoacoustic (PA) signal intensity of a line of oil-based ink on white office paper. For the black, green, and blue ink, the wavelength dependence of the PA signal intensity was measured. No PA signal was detected for either the unmarked office paper or the location of the red ink.

With the PAI-05 system, the wavelength could be arbitrarily selected from 750 to 850 nm. Figure 8a shows the reflectance spectral characteristics of the white office paper and four colors of oil-based ink colors (black, red, green, blue) written on the paper. Figure 8b shows the wavelength dependence of the PA intensity obtained using the PAI-05 system. As the light intensity reflected by the sample was small, the light absorbance was increased, which resulted in increased PA intensity. This is a valid result as a PA property. No PA signals were obtained from the white office paper or red ink at all.

### PAI of a human subject

**Figure 9.**
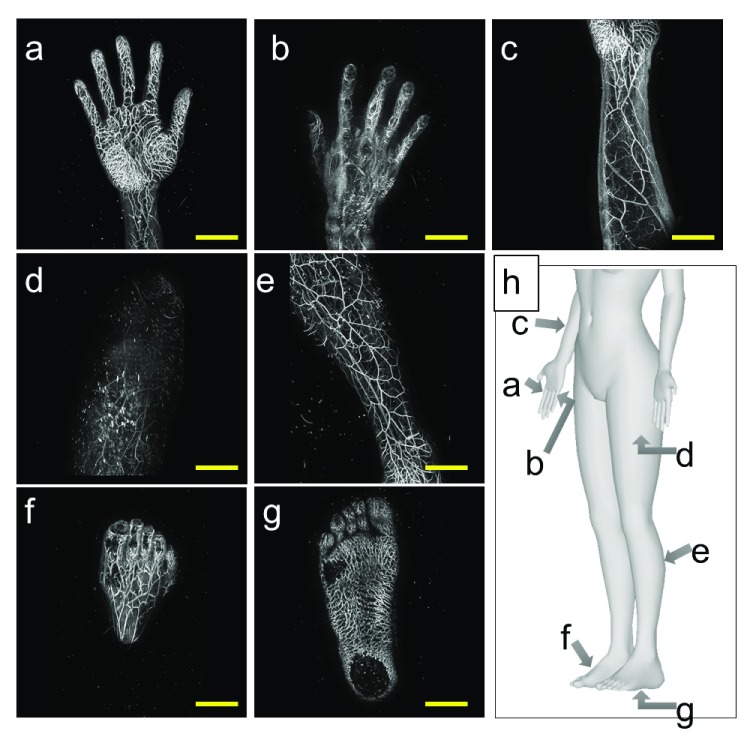
Photoacoustic (PA) images obtained by the PAI-05 system of various extremities. ( **a**) Palm. ( **b**) Back of the hand. ( **c**) Forearm. ( **d**) Anterolateral thigh (ALT). ( **e**) Lower thigh. ( **f**) Top of the foot. ( **g**) Sole of the foot. ( **h**) Schematic diagram showing the locations of Figures 9a–g.

Next, the living subject was imaged. This system was newly designed to facilitate the imaging of limbs. We designed the holding tray to be shallower and wider than before to make it more comfortable for a subject to assume the posture required for scanning. Figures 9a–g are examples of PA images of the extremities of a living body. It was confirmed that PA images in a wide range of 270 mm × 180 mm could be obtained in each area.

Similar to the results of the phantom experiments, as shown in Figure 9a and other figures, PA images of blood vessels could be reconstructed with definition equal to or higher than that of the previous prototypes ^6– 8^.

In the image of the sole of the foot shown in Figure 9f, the PA image of blood vessels in the heel region was not recognized as a network shape, unlike the surroundings. This may be because body weight was applied to the heel at the time of measurement, thus inhibiting blood flow in the subcutaneous veins.

Figure 9g shows a PA image of the dorsum of the foot. The whole image of the region could not be shown because the whole dorsal foot was not immersed in water in the shallow holding tray. Likewise, the upper and lower parts of the lower leg shown in Figure 9e were not visualized because they protruded from the water.

The thigh shown in Figure 9d was shaved in advance, but black hair roots remained, and the signal intensity of these roots was stronger than that of the blood vessels. Therefore, the image produced by removing the hair volume data using cloth simulation is shown.

**Figure 10.**
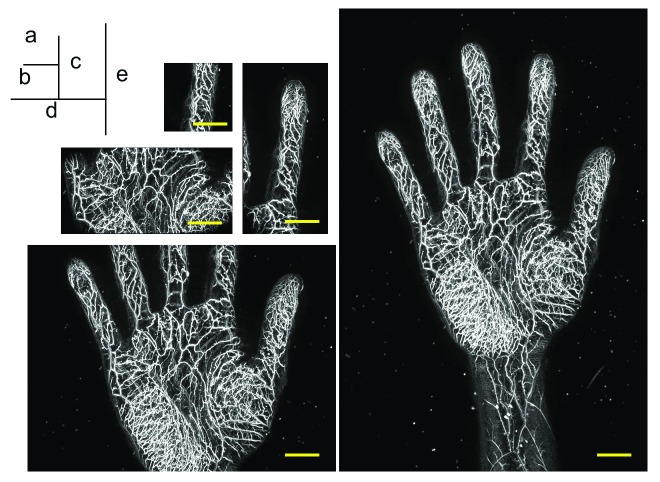
Various PA images obtained in still mode for different scanning areas. ( **a**) Image 40 * 40 mm in the *x*- *y* direction. ( **b**) Image 100 mm in the *x*-direction * 50 mm in the *y* direction. ( **c**) Image 50 mm in the x-direction * 100 mm in the *y* direction. ( **d**) Image 180 mm in the *x*-direction * 135 mm in the *y* direction. ( **e**) Image 180 mm in the x-direction * 270 mm in the *y* direction. The yellow bar represents 20 mm in all figures.

Figures 10a–e show examples of images acquired using different scanning areas in still mode. The maximum still image area of 270 mm × 180 mm ( Figure 10e) was imaged in approximately 10 minutes, and the minimum still image area of 40 mm × 40 mm ( Figure 10a) was imaged within one minute, as shown in Table 1.

**Figure 11.**
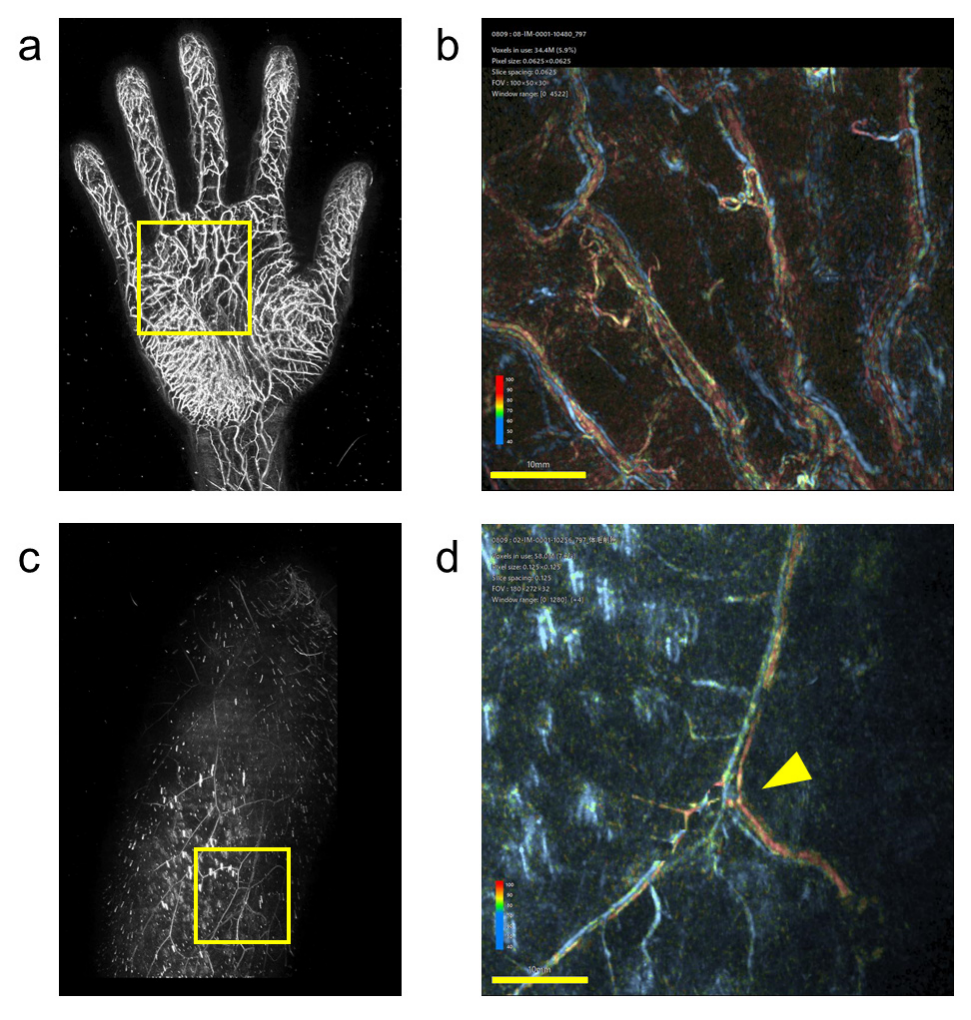
Example of S-factor image calculated from two-wavelength photoacoustic (PA) images. ( **a**) Gray scale image of the palmar. Yellow square suggests the area of calculation of S-factor shown in Figure 11b. ( **b**) Examples of common palmar digital arteries and their accompanying veins. ( **c**) Gray scale image of whole anterolateral thigh (ALT) image. Yellow square suggests the area of calculation of S-factor shown in Figure 11d. ( **d**) Artery and vein in the ALT. Yellow arrows suggest blood vessels considered perforators.

Figure 11 shows S-factor images of the palms and thigh at 756 and 797 nm under the same conditions as in previous papers ^5, 10, 15^ using alternating irradiation from two different lasers ^10^. A PA image showing blood vessels that could be distinguished as an artery or vein was obtained. Figures 11a–b show images of blood vessels in the palm. Figure 11a shows a total MIP image including the skin surface, and 11b shows a MIP image of the deep region after deletion of the subcutaneous vein network. It is generally known that an artery is accompanied by one or two vein(s). Such accompanying blood vessels near the common palmar digital arteries could be visually recognized from these figures that by their different S-factor values. Figures 11c–d show images of the blood vessels in the ALT. Figure 11c shows a total MIP image including the skin surface, and Figure 11d shows a MIP image of the deep region after deletion of the surface image of hair roots, skin melanin, and subcutaneous vein network. As with the image of the palm, it was visually recognized that both arteries and veins were present side by side.

**Figure 12.**
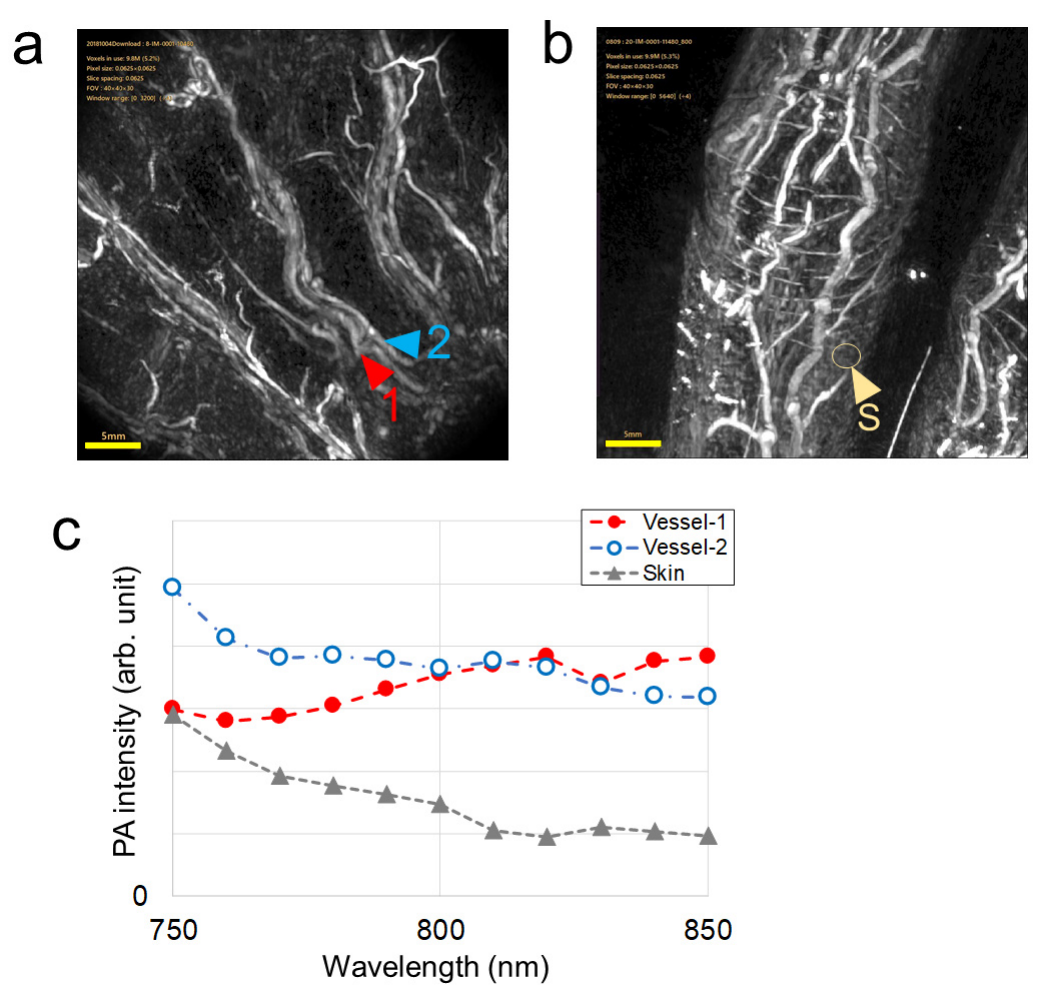
Example of the dependence of photoacoustic (PA) signal intensity in living tissue on wavelength. ( **a**) The results of an artery and a vein. ( **b**) The result of skin melanin. ( **c**) Each PA intensity as a function of the wavelength.

In the PAI-05 system, two laser wavelengths can be arbitrarily selected within the range of 750 to 850 nm. Stable S-factor images were obtained by adopting alternating irradiation. Figures 12a–b show the area used for analyzing PA intensity for different wavelengths. Figure 12c is an example of the analysis of the signal intensity of each region, showing dependence of the wavelength on each component of the blood vessels and melanin.

**Figure 13.**
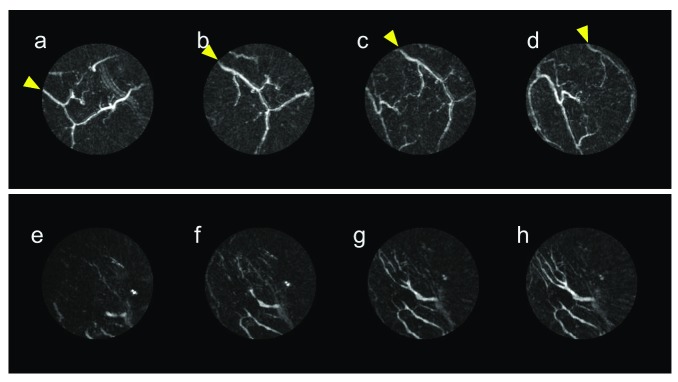
Snapshots of the motion pictures acquired in movie mode. ( **a**) Snapshots from the motion pictures of a hand being swung left and right in the horizontal plane ( Supplementary Video 4). Yellow arrows indicate the same blood vessels. ( **b**) Snapshots from the motion pictures of blood flow changing when a fingertip is pressed against the tray and then released ( Supplementary Video 5).

Figures 13a–h show snapshots captured in movie mode within an FOV 2 cm in diameter. Figures 13a–e are snapshots of a motion picture ( Supplementary Video 4) captured while the palm was moving left and right. Figures 13f–h are snapshots of motion pictures captured while the subject was pressing a finger onto the bottom of the tray; immediate changes in blood flow can be observed when the pressure was released ( Supplementary Video 5). Although motion pictures captured by PAI have previously been reported, the effective the FOV of each individual volumetric frame was smaller than that of our system ^4^. Our system could provide more extensive motion pictures.

Zip file containing the underlying data of the presented results in excel files Figure 3 – Laser irradiation profile data (Fig3_LaserProfile) Figure 6c - Line profile of photoacoustic (PA) intensity data (Fig6c_LSF) Figure 6e – Contrast profile data (Fig6e_ContrastProfile) Figure 6g – Photoacoustic (PA) intensity for each phantom with different angles (Fig6g_oblique) Figure 7e – Movie photoacoustic (PA) intensity data (Fig7e_MoviePAIntensity) Figure 8a - Reflectance spectra data for oil ink on white office paper (Fig8a_Spectrum_OilPen) Figure 8b – Photoacoustic (PA) signal intensity data for oil ink on white office paper (Fig8b_PAoil-ink) Figure 12 – Signal intensity data from clinical samples (Fig12_ClinicalSignalIntensity) Click here for additional data file. Copyright: © 2019 Nagae K et al. 2019 Data associated with the article are available under the terms of the Creative Commons Zero "No rights reserved" data waiver (CC0 1.0 Public domain dedication).

## Discussion

In the PAI-05 system, the HDA consisted of 1024 high-density sensors arranged inside a hemispherical casing with a diameter of 110 mm.

In the previous designs ^3, 5– 10^, approximately 500 discrete rod-shaped sensors were inserted into a hemispherical casing with a diameter of 254 mm, but designing such a high-density arrangement for this study might have been difficult with conventional methods. The HDA design developed for this study using film-type sensors has the potential to greatly simplify the sensor manufacturing process for practical use because it was able to realize a 1024-channel arrangement, which may be difficult with discrete elements. This design made it possible to obtain images with less noise even with one shot, contributing to the realization of real-time motion pictures.

While real-time motion pictures in PAT have mainly been reported in combination with conventional B-mode ultrasound ^16, 17^, the reproducibility of the blood vessel morphology in the PA images seems to be poor. Although the resolution seems to be good in the plane of a previously reported ring sensor ^24^, the resolution in the direction normal to the ring surface is reportedly several mm or more. 3D structural analysis using images with such large anisotropy would be difficult.

As shown in Figure 6 and in other papers ^3– 10^, PA images obtained by a PAI system with an HDA show nearly isotropic spatial resolution, which is considered optimal for reproducing the morphology of blood vessels. Information for diagnosis can be obtained by performing 3D observations from different directions using not only still image but also real-time motion pictures. There is no doubt that larger imaging areas for capturing both still images and motion pictures will greatly contribute to the diagnosis of conditions in living organisms.

Regarding the phantom evaluation, sufficient contrast was obtained at a depth of 2 cm in still mode. This depth is nearly sufficient for imaging the subcutaneous vessels of limbs. Linear-type phantoms could be imaged from 0 to 70 degrees with respect to the evaluation of the “limited view problem,” so it can be expected that most actual blood vessels in the living subject can be observed using the PAI-05 system. On the other hand, there is still an issue that perforators rising from deep regions may be difficult to visualize, as noted in a previous paper ^7^. Clinical research in biological organisms should be continued, and diagnostic capability of the system should be verified.

In the phantom experiment for evaluating the image quality in movie mode, the noise could be reduced by increasing the number of images averaged (N), as shown in Figure 7. Generally, the amount of noise is proportional to $1/\sqrt{N}$ in the case of white noise. The noise improvement effect in SM mode was smaller than $1/\sqrt{N}$, suggesting that the noise was not a random system noise. As shown in the upper row of Figure 7f, the shape of the noise showed good reproducibility. Therefore, it is suggested that it was a fixed artifact pattern. In FM mode, the position of the HDA changes with each laser irradiation. The spatial distribution of artifacts is changed by shifting the relative position of the HDA with respect to the absorber. Artifacts in FM can be suppressed by averaging the various spatial distributions. It seems that both the system noise and the effect of the artifact were reduced by increasing N, as shown in the lower row of Figure 7f. Because this suppression effect was in accordance with $1/\sqrt{N}$ up to N=10, for which the position of the probe varied, it can be considered that the noise suppression effect was realized by superimposed data in an almost uncorrelated spatial state.

As for the evaluation of the living subject, it was possible to appropriately scan the limbs by designing the system configuration to be suitable for limb imaging. Since it is now possible to obtain PA images in a wide range, it is expected that diagnosing conditions affecting blood vessel will be made easier by viewing more complete images. These images will also be able to provide clinically useful information, such as that necessary for preoperative planning.

Adopting a rectangular spiral scan instead of the conventional circular spiral ^3, 5– 10^ could not only reduce the dead space when scanning long body parts but also shorten the scanning time.

Nonetheless, because the scanning time varies depending on the size of the area when applied for routine clinical diagnosis, physicians may need to carefully determine the minimum required scanning range in consideration of the state of the patient.

The PAI-05 system is limited to four scanning modes, as shown in Table 1; however, if it becomes possible to freely select the region of interest (ROI) according to the shape of the subject, to the scanning time could be further shortened.

There were body parts that could not be imaged as they were protruding from the water in the holding tray. This may be a large problem to be solved, especially in the context of scanning elderly patients or areas that require an awkward posture. Another approach for acoustic matching between the subject and the holding tray, such as applying an acoustic matching gel ^15^, may have to be considered.

The ability to arbitrarily select the wavelength may contribute to improving the quantitative analysis of SO _2_^25, 26^. This capability could also enable the imaging of externally added dyes, such as indocyanine green (ICG), and thus enhance the applicability of PAT ^27^. There are still no useful clinical application based on the clinical evidence of real-time 3D PAI, but new clinical applications of our PAI system will be developed and are expected to be proven in the near future. For example, diagnostic imaging using molecular probes is currently under development for future applications. Additionally, high-definition 3D imaging modes could potentially clarify drug delivery characteristics with high temporal resolution. The potential applications of the PAI-05 system reported here could be considered great advances in PAT technology. Clinical research in patients will be conducted in the future. The device performance for actual human subjects will be evaluated at that time.

Clinical research in patients will be conducted in the future. A high-quality 3D motion picture (so-called 4D imaging) with submillimeter resolution may be difficult even with CT or MRI, and it is expected that new clinical findings will be obtained with the PAI-05 system. This system is promising for obtaining a wide range of high-definition images not only for the preoperative planning of free flap surgery but also for the PAT-based diagnosis of breast cancer, as previously reported ^10^.

In summary, we developed a new system, named PAI-05, dedicated to limb imaging and showed its properties in phantom experiments. We also imaged the limbs of a living subject in a wide range using the PAI-05 system. As with the conventional prototype, a high-resolution arteriovenous image was obtained by label-free imaging. Real-time motion pictures in an area with a diameter of 20 mm could be obtained. We expect to develop new clinical applications for the new PAT system.

## Data availability

The data referenced by this article are under copyright with the following copyright statement: Copyright: © 2019 Nagae K et al.

Data associated with the article are available under the terms of the Creative Commons Zero "No rights reserved" data waiver (CC0 1.0 Public domain dedication). 

F1000Research: Dataset 1. Zip file containing the underlying data of the presented results in excel files, https://doi.org/10.5256/f1000research.16743.d224869 ^28^

Description of content

Figure 3 – Laser irradiation profile data (Fig3_LaserProfile)

Figure 6c - Line profile of photoacoustic (PA) intensity data (Fig6c_LSF)

Figure 6e – Contrast profile data (Fig6e_ContrastProfile)

Figure 6g – Photoacoustic (PA) intensity for each phantom with different angles (Fig6g_oblique)

Figure 7e – Movie photoacoustic (PA) intensity data (Fig7e_MoviePAIntensity)

Figure 8a - Reflectance spectra data for oil ink on white office paper (Fig8a_Spectrum_OilPen)

Figure 8b – Photoacoustic (PA) signal intensity data for oil ink on white office paper (Fig8b_PAoil-ink)

Figure 12 – Signal intensity data from clinical samples (Fig12_ClinicalSignalIntensity)

## Supplementary materials

Supplementary Video 1: **Motion picture showing the PAI-05 system configuration and the principle of data acquisition.**

Supplementary Video 2: **Motion picture showing the scan sequence of the still mode of the PAI-05 system.**

Supplementary Video 3: **Motion picture showing the scan sequence of the fluctuation movie mode of the PAI-05 system.**

Supplementary Video 4: **Motion picture of a hand being swung left and right in the horizontal plane.**

Supplementary Video 5: **Motion picture of blood flow changing when a fingertip is pressed against the tray and then released.**
