# Supplementary material for: Real-time 3D Photoacoustic Visualization System with a Wide Field of View for Imaging Human Limbs
Source: F1000Res. 2019 Feb 7;7:1813. Originally published 2018 Nov 19. [Version 2] doi: 10.12688/f1000research.16743.2 (PMC6396844; doi:10.12688/f1000research.16743.2)
Supplement: Peer review history

# Peer review: Reviewer response for version 2

**DOI:** 10.5256/f1000research.19817.r44428

**Published:** 2019-02-28

**Authors:** Jun Xia

**Affiliations:** 1 Department of Biomedical Engineering, University at Buffalo, State University of New York, Buffalo, NY, USA

No further comments.

I have read this submission. I believe that I have an appropriate level of expertise to confirm that it is of an acceptable scientific standard.

# Peer review: Reviewer response for version 1

**DOI:** 10.5256/f1000research.18301.r41248

**Published:** 2018-12-28

**Authors:** Hideyuki Hasegawa

**Affiliations:** 1 Graduate School of Science and Engineering, University of Toyama, Toyama, Japan

This paper describes a state-of-the-art system, which is capable of real-time photoacoustic imaging of a living human body. The authors evaluated fundamental characteristics of the system, such as spatial resolution and angle-dependent visibility of a target, and also showed the feasibility of the system in in vivo imaging of vascular trees in a living human body. The system is able to visualize small vessels with good resolution and contrast. The contents of the paper would be of interest for the readers. Upon indexing, I suggest revisions described below:

  

1. There are number of referenced papers published from authors' group. On the other hand, the papers from other research groups seem few. Are there no more related works from other research groups?
2. Although the method for image reconstruction is described in [21], it would be better to describe how to do that briefly. Particularly, the diverged laser light has a significant spatial inhomogeneity as shown in Fig. 3. Can you obtain an image by one shot of laser pulse with such a spatial inhomogeneity? Using such a diverging light source, photoacoustic signals would also be spatially inhomogeneous. Do you need any correction compensating for such a spatial inhomogeneity in the light intensity?
3. It would be more informative if you describe the reason for the use of fluctuation of the light source in the fluctuation mode (FM). Does the fluctuation change the spatial distribution of artifacts? If so, it is understandable that image quality is improved by averaging.
4. The size of an ultrasonic transducer element is relatively large (2 mm). I understand that the sensitivity of each transducer element should be high, but are there any effect of the element directivity on image quality, such as artifact generation?
5. In p. 7 and l. 2 in the right column, epsilon_Delta Hb should be epsilon_Hb?
6. In p. 7 and l. 3 in the right column, I found an expression "epsilon_Delta Hb and is". "and" should be removed?
7. As explained in the paper, the scan time increases with widening the field of view. Do you need any motion compensation technique because the spatial resolution is very high?
8. In the case of alternative laser irradiation, are there any effects of movements of blood cells during the repetition period of 50 ms? In the in vivo experimental results, relatively large vessels are also visualized, and movements of blood cells in such a relatively large vessel would be significant owing to relatively high flow velocity.
9. In Fig. 6b, I can see some artifacts. Such a distribution of the artifacts (sidelobes?) depends on the spatial arrangement of array elements based on the Fibonacci grid? It would be better to describe why artifacts can be suppressed by the Fibonacci grid and how large the sidelobe level is when a regular grid is used.
10. Particularly in Fig. 6e, I can see remarkable range (y-direction) sidelobes. What is the source of those sidelobes? Also, such sidelobe components do not affect the estimation of oxygen saturation?

I have read this submission. I believe that I have an appropriate level of expertise to confirm that it is of an acceptable scientific standard.

# Response

**Published:** 2019-02-05

**Authors:** Yoshiaki Matsumoto

**Affiliations:** Kyoto University Hospital, Japan

Dear Prof. Hideyuki Hasegawa (Reviewer #2)

The authors would like to thank Reviewer #2 for your decision of “Approved". In order to aim more high-quality paper, we responded to the comments of Reviewer # 2 and made corrections as necessary

(1)    Response: Not revised

As well-known, there are many papers on PAT, but there are not many papers as for PAT with HDA, especially for actual clinical use. Although there are various system configurations such as linear probe method, method using Fabry Perot, ring-shaped method and so on, there are too many to describe all other methods in a well-balanced manner. For this reason, we focused on HDA-PAT system and its clinical use. Based on this policy, we did not add the other references.

(2)    Response: Agreed

Your point is right. When calculating the absorption coefficient from the initial sound pressure, it is necessary to correct the spatial non-uniformity of the light intensity including the in-vivo depth direction.

On the other hand, if only image evaluation is performed qualitatively, it is not always necessary to correct the light intensity. This is because, in the Still mode, spatial nonuniformity of light intensity is corrected by averaging images obtained by scanning the hemispherical sensor in a wide range.

In the Movie mode, since the light nonuniformity cannot be compensated, the signal may become dark around the image. But we can evaluate the image qualitatively. We revised the following sentences.

(Before)

We used universal back-projection (UBP) [21] for PA image reconstruction.

(After)

We used universal back-projection (UBP) [21] for PA image reconstruction. When calculating the absorption coefficient from the initial photoacoustic pressure, it is necessary to perform the light intensity correction. In this paper, however, we did not carry out it and limited the discussion within the qualitative consideration.

(3)    Response: Agreed

 Your point is right. We revised the following sentences.

(After)

The spatial distribution of artifacts is changed by shifting the relative position of the HDA with respect to the absorber. Artifacts in FM can be suppressed by averaging the various spatial distributions.

(4)    Response: Not revised

We are reconstructing a spatial region with high receiving sensitivity of photoacoustic waves specified by element size and element placement. Since the image reconstruction range is limited, we have confirmed the influence of artifacts is not large.

On the other hand, if the element size is smaller, image reconstruction in a wider spatial region becomes possible. However, it is necessary to expand the light irradiation region. In that case, there is a possibility that the artifact increases due to increase of artifact sources. We made no additional sentence.

(5)    Response: Agreed

 Your point is right. We revised the following sentences.

(Before)

ε∆Hb is the molar extinction coefficient of deoxyhemoglobin;

(After)

εHb is the molar extinction coefficient of deoxyhemoglobin;

(6) Response: Agreed

 Your point is right. We commented on the docx file.

(7)    Response: Agreed but no revised

Yes. We consider that the motion compensation technique can derive a better image, but it is not utilized in this paper.

We can do it as written in the previous article, so we will adopt it as necessary in future clinical research. We made no additional sentence.

(8)    Response: Not revised

 With the spatial resolution of the current device, we cannot discriminate red blood cells individually, so we believe there is no effect of blood flow. We made no additional sentence.

(9)    Response: Not revised

The artifacts seen in Fig. 6 are generated not by arrangement of the elements but by the discontinuation of the detectors around edges of the HDA.

The effectiveness of adopting Fibonacci grid is to suppress the artifacts generated in a specific direction since the detectable area of each element is uniform. We made no additional sentence and quantitative arguments.

(10)    Response: Not revised

 It is also an artifact due to the discontinuation of the detectors around edges of the HDA as same as the (9). Oxygen saturation of blood vessels overlaid on this artifact may be affected by artifacts. We made no additional sentence.

Best regards,

Kenichi Nagae and Yasufumi Asao

# Peer review: Reviewer response for version 1

**DOI:** 10.5256/f1000research.18301.r41068

**Published:** 2018-12-06

**Authors:** Jun Xia

**Affiliations:** 1 Department of Biomedical Engineering, University at Buffalo, State University of New York, Buffalo, NY, USA

This manuscript demonstrates performance of the latest photoacoustic system from Canon Inc. The images are very impressive and definitely showcase the potential of photoacoustic imaging for clinical applications. Compared to the previous generation, major improvements in the current system include the use of a 1024-element transducer array (vs. 512 elements), implementation of dual wavelength imaging, a flat bed for limb imaging, and dynamic imaging of motion. Overall the manuscript is well written and the study is comprehensive.

 The study could be further improved in the following aspects:

1. Please quantify the imaging depth in human. Calculation in phantom is not sufficient as the phantom composition could not precisely represent the optical attenuation of human tissue.
2. Application of the system is unclear and needs to be clarified. The palm images could be used for biometric application as mentioned in a previous publication ^1^. Could the system be miniaturized for that application? Foot imaging could be used for chronic ulcer assessment. However, most of those patients are immobile and the imaging pose may not be ideal for these patients.
3. The authors suggested ICG imaging. For that application, photoacoustic imaging will compete with fluorescence imaging, such as the SPY system from Novadaq. What would be the pros and cons of PAI in comparison to a clinical fluorescence imaging system?
4. Please indicate the magnitude of amplification in the data acquisition system. 
5. The original system from Dr. Kruger's group used a 128-element hemispherical array ^2^. The system has been improved over years to 1024 elements. Is there a cut-off number, beyond which further increase in element number will not improve the image quality? 
6. After reconstruction, did the authors use any image processing techniques to enhance vascular structures?

I have read this submission. I believe that I have an appropriate level of expertise to confirm that it is of an acceptable scientific standard, however I have significant reservations, as outlined above.

## References

[1] WangYLiZVuTNyayapathiNOhKXuWXiaJ: A Robust and Secure Palm Vessel Biometric Sensing System Based on Photoacoustics. *IEEE Sensors Journal*.2018;18(14) : 10.1109/JSEN.2018.2843119 5993-6000 10.1109/JSEN.2018.2843119

[2] KrugerRALamRBReineckeDRDel RioSPDoyleRP: Photoacoustic angiography of the breast. *Med Phys*.2010;37(11) : 10.1118/1.3497677 6096-100 10.1118/1.3497677 21158321PMC2994935

# Response

**Published:** 2019-02-05

**Authors:** Yoshiaki Matsumoto

**Affiliations:** Kyoto University Hospital, Japan

Dear Prof. Jun Xia (Reviewer #1)

The authors would like to thank Reviewer #1 for your detail comments and suggestions. It is a great honor to receive high evaluation. However, if there is concern about duplication with the results and discussion of clinical research papers planned to be submitted in the future, we cannot describe that point deeply. Therefore, there may be points that are not answered enough for comments.

(1)    Response: Partially agreed

We agree that the phantom's evaluation is inadequate as you pointed out, and evaluation by the human being is indispensable for showing the device performance. Nonetheless, we think that it is not good data to quantify it with just one healthy person this paper. There is no guarantee that this person is typical.

The results of clinical research using the device disclosed this time will be papers in future. The depth performance of human imaging should be discussed in multiple cases among them. So, we added the following sentence to the discussion section.

(Additional sentence)

Clinical research in patients will be conducted in the future. The device performance for actual human subjects will be evaluated at that time.

(2)    Response: Agreed but not revised

The applicability to biometric authentication has already been mentioned in reference [6], so it is omitted in this paper.

In this paper, we designed the device that can measure limbs in a versatile medical usage. It is primarily targeted for medical applications and has been evaluated as useful for research on plastic surgery area using this device. Through clinical research, issues for patients have come to be seen and we are aware that the current situation is not ideal as you pointed out. Therefore, it is described as follows.

“This may be a large problem to be solved, especially in the context of scanning elderly patients or areas that require an awkward posture. Another approach for acoustic matching between the subject and the holding tray, such as applying an acoustic matching gel [15], may have to be considered.”

It is no doubt that it can be downsized by technical development, but we have not confirmed details, so I think that it is not appropriate to describe as a paper.

Further clinical issues to be solved should be listed in individual clinical papers to be submitted in the future.

We would like to maintain the text from the original paper without any additional sentence.

(3)    Response: Agreed but not revised

We also started clinical studies using ICG imaging, and also presented the results of comparison between SPY (or PDE) and PAI at domestic and international conferences. It will be appeared in a clinical paper shortly. Since conflicts are concerned, we would like to avoid detailed description. 

(4)    Response: Agreed

We added the sentence to the device configuration section.

(Additional sentence)

We set the amplification rate to 48 dB in this study.

(5)    Response: Agreed but not revised

We calculated it and we concluded that until the densest sensor arrangement on the hemispherical array, the image quality, which corresponds the number of artifacts, monotonically improves in proportion to the number of sensors. In other words, there is no cutoff number.

We did not modify the text for this comment.

(6)    Response: Not revised

No special image processing has been done. We made no additional sentence.

Best regards,

Kenichi Nagae and Yasufumi Asao
